# Shannon's Energy Based Algorithm in ECG Signal Processing

**DOI:** 10.1155/2017/8081361

**Published:** 2017-01-18

**Authors:** Hamed Beyramienanlou, Nasser Lotfivand

**Affiliations:** Department of Electronic Engineering, Islamic Azad University, Tabriz Branch, Tabriz, Iran

## Abstract

Physikalisch-Technische Bundesanstalt (PTB) database is electrocardiograms (ECGs) set from healthy volunteers and patients with different heart diseases. PTB is provided for research and teaching purposes by National Metrology Institute of Germany. The analysis method of complex QRS in ECG signals for diagnosis of heart disease is extremely important. In this article, a method on Shannon energy (SE) in order to detect QRS complex in 12 leads of ECG signal is provided. At first, this algorithm computes the Shannon energy (SE) and then makes an envelope of Shannon energy (SE) by using the defined threshold. Then, the signal peaks are determined. The efficiency of the algorithm is tested on 70 cases. Of all 12 standard leads, ECG signals include 840 leads of the PTB Diagnostic ECG Database (PTBDB). The algorithm shows that the Shannon energy (SE) sensitivity is equal to 99.924%, the detection error rate (DER) is equal to 0.155%, Positive Predictivity (+P) is equal to 99.922%, and Classification Accuracy (Acc) is equal to 99.846%.

## 1. Introduction

**Figure 1:**
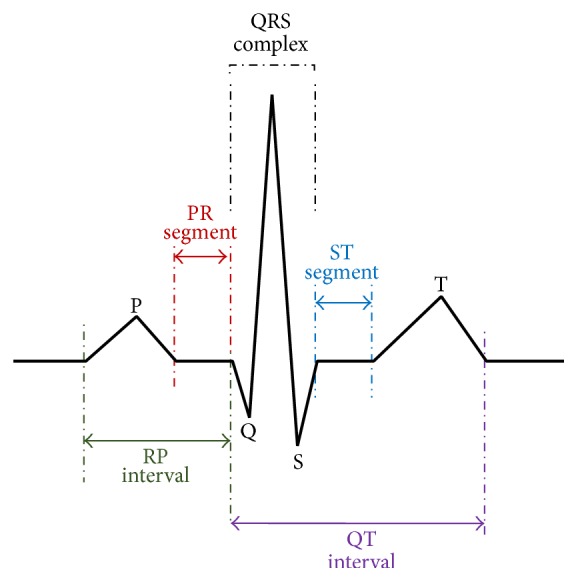
Schematic diagram of normal ECG.

In recent years, cardiovascular disorders have been one of the major diseases threatening human life. Therefore, the detection of heart signal waves such as QRS complex is highly significant [1]. Electrocardiogram is used to detect most of heart disorders and shows the electrical activities of heart as a signal [2]. ECG signals contain a lot of information concerning heart diseases. The detection of special points and different parameters such as QRS complex are one of the basic topics and are of high importance, because they lead to the diagnosis of heart diseases. The QRS are used to diagnose many cardiac diseases and noncardiac pathologies such as autonomic malfunction vascular, respiratory (RR) assessment in cardiomyopathy and the normal ventricular myocardium, estimate the heart rate and heart rate variability analysis, and detect ST segment [3–5]. Heart problems usually involve leaking valves and blocked coronary arteries. This research is motivated by reasons expressed. Heart rate cycle consists of a P-wave, a QRS complex, T-wave, and sometimes U-wave [5].  Figure 1 shows schematic representation of normal ECG.

Detecting any of heart signal waves may be difficult due to variable physiology, arrhythmia, disease, and noise. Therefore, in methods such as artificial neural networks and supportive vector machines, detection by the wave R is not always successful and true detection cannot be reached in different signals [6, 7].

The shape of the waves T, P, and QRS is well known; however, the time and frequency of these waves depend on the physiological and physical conditions. In addition, the signal may face polluted recordings with noises such as transmission lines [3].

In recent decades, various methods have been presented to improve the detection of heart signal waves, including Pan-Tompkins algorithm [7], Wavelet Transform, by usage of a constant scale in signal analysis, not considering the characteristics of the signal [8, 9], and artificial neural networks, containing of a series of interconnected simple processing units that each connection has a weight. Input layer, one or multiple hidden layers, and output layer constitute a neural network [10, 11]. Adaptive filter [12], called Hilbert-Huang Transform (HHT), is a new technique for extracting features that are nonlinear and nonstationary signals. This technique has a leakage in practical tasks [13]. Filter bank [14], a Hidden Markov Model (HMM), describes the process where direct observation is not possible, when sequence of symbols can observe HMM. It is used in many fields such as classification of heartbeat and apnea bradycardia detection in preterm infants [15]. Hermite Transform (HT) was recently used instead of Fourier Transform. HT shows better performance, when optimization is done properly [16]. Threshold method [17], Shannon energy envelope (SEE), is the average spectrum of energy and is better able to detect peaks in case of various QRS polarities and sudden changes in QRS amplitude. SEE detects R-peak with a better estimate [18]. S-Transform and Shannon energy (SSE) create a frequency-dependent regulation which is directly related with the Fourier spectrum. S-Transform includes short time Fourier Transform (STFT) and the Wavelet Transform (WT). SSE gives a smooth cover for P-waves and T-waves and completely decreases their influence [19]. Methods such as pattern matching are based on their comparing and contrasting. The calculations are complex and need manual classification [6].

In this paper, an algorithm based on Shannon energy has been proposed to improve the QRS complex detection and simplify the detection process. First, a band-pass filter is used for eliminating noise. Second, Shannon energy of ECG signal is calculated. Third, include moving averages and a differential for the envelope of step 2. Finally, with defining a threshold, peaks are detected. The proposed algorithm is tested on 115-second (to end) ECG signal of PTB Diagnostic ECG Database (PTBDB) [20, 21] and detection accuracy of 99.846% is obtained. The proposed technique results in good performance without being mathematically complex.

## 2. Method

**Figure 2:**
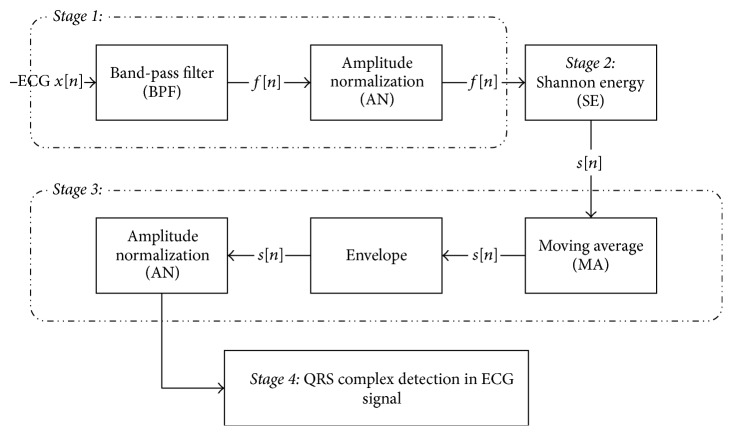
Block diagram to detect QRS complex.

The block diagram of detecting QRS complex algorithm is shown in Figure 2. It includes four stages. Stage 1 includes band-pass digital filter and amplitude normalization. Stage 2 includes calculating Shannon energy of stage 1. In stage 3, with moving average and differencing, make a pack of Shannon energy, and in stage 4, with defining a threshold, QRS complex is detected.

### 2.1. Preparations Signal

Digital-analog conversion process is causing all kinds of noise interference and sometimes strongly affects the information. These interactions include frequency interference, muscle contraction, and wandering signals from the baseline or Gaussian white noise [5].

The ECG signal recorded from human beings is a poor signal and is often contaminated by noise. Frequency interference includes a narrow band from 48 to 60 Hz and harmonic interference, and the noise from muscle contraction occurs in 38 to 45 Hz. To eliminate this noise, notch filter is good [22]. Deep breathing, loosely connected electrodes, and sudden changes in voltage lead the baseline signal to be wondered (baseline drift) [5]. Random variable vector (mean) and chromatogram baseline estimation and denoising using sparsity (BEADS) algorithm [23] are good methods to eliminate baseline drift. The band-pass filter decreases efficacy of muscle contraction, frequency interference, baseline drift, and P-wave and T-wave interference [7, 24]. To repress these noises, Butterworth band-pass digital filter with stop-point set at 5 to 16 Hz is used. Butterworth has no ripple in band-pass. [25]. After band-pass filter, the signal is normalized with (1) in stage 1 [26].

(1) $$\begin{array}{c} a\left[\;n\;\right]=\frac{\left|f\left[n\right]\right|}{max_{i=1}^{N}\left|f\left[n\right]\right|}, \end{array}$$

where *a*[*n*] is a normalized amplitude; *f*[*n*] is an after processes band-pass filter (BPF). *N* denotes the number of samples.

### 2.2. Shannon Energy and Detection of QRS Complex

The proposed method is based on the use of signal energy. The signal square is very close to the signal energy. For discrete time signal energy is defined as follows:

(2) $$\begin{array}{c} E_{x}=\overset{\infty }{\underset{-\infty }{\sum }}x\left(\;n\;\right)x^{*}\left(\;n\;\right)=\overset{\infty }{\underset{-\infty }{\sum }}{\left|\;x\left(\;n\;\right)\;\right|}^{2}. \end{array}$$

Here, *E*_*x*_ expresses the signal energy, *x*(*n*) defined ECG data, and *n* is samples. ∑ represents sum from (−*∞*  *∞*) [27]. To explain, we have the following:

(3) $$\begin{array}{c} E_{x}=\left(\;x_{0}^{2}+x_{1}^{2}+x_{2}^{2}+x_{3}^{2}+\cdots \;\right). \end{array}$$

Shannon energy calculates the average spectrum of the signal energy. In other words, discount the high components into the low components. So, input amplitude is not important. Shannon energy and Hilbert Transform (SEHT) provide a good accessory for detecting R-peak but this technique has a problem. SEHT needs high memory and has delays [28]. It is designed for solving our actual requirements. To find smooth Shannon energy, zero-phase filter and Shannon energy approximate are playing a basic role [24, 28].

Shannon energy (SE) calculates the energy of the local spectrum for each sample. Below is a calculation of Shannon energy:

(4) SE=−anlog⁡an,sn=−a2nlog⁡a2n,

where *a*[*n*] is after process normalization.

Energy that better approaches detection ranges in presence of noise or domains with more width results in fewer errors. Capacity to emphasize medium is the advantage of using Shannon energy rather than classic energy [18, 19]. The selected signal is normalized with (5) in stage 3 for decreasing the signal base and placing the signal below the baseline.

(5) $$\begin{array}{c} s\left[\;n\;\right]=\frac{s\left[n\right]-\mu }{\sigma }, \end{array}$$

where *μ* is the random variable vector and *σ* defined standard deviation of the signal.

In stage 3, after computing Shannon energy, small spikes around the main peak of the energy are generated. These spikes make main peaks detection difficult. To eliminate this spike, Shannon energy is converted into energy package (Shannon energy envelope (SEE)). To overcome this problem, the Hilbert Transform is used. SEHT method is a simple and high accessory but the SEHT needs high memory and has delays, so it is unfit for real time detection [24, 28]. To smooth out the spikes, rectangular (*h*) with *L* length is used. Filtering operation is shown as follows:

(6) mn=filter h,j,S,m′n=filter h,j,S,

where *m*[*n*] defines moving average, *j* is a constant, and *S* defines Shannon energy from previous steps. For spikes reducing and enveloping, the nonzero peaks obtained from differential get linked. In other words, diagnosed peaks are linked together.

Difference is defined below:

(7) $$\begin{array}{c} d\left[\;n\;\right]=f\left[\;n\;\right]-f\left[\;n-1\;\right],\;n=2,3,\ldots . \end{array}$$

The sign is defined as follows:

(8) $$\begin{array}{c} sgn\left(\;x\;\right)\left\{\begin{array}{cc} -1 & \text{if }x<0\\ 0 & \text{if }x=0\\ 1 & \text{if }x>0, \end{array}\right. \end{array}$$

where *x* is a real number.

In stage 4, positive peaks are QRS complex location. To detect QRS complex, a threshold (see (9)) is defined. In fact, samples with greater amplitude than the threshold are selected as output.

(9) threshold=κμ1−σ2if σ<μ,threshold=κσ1−μ2if σ>μ,

where *κ* is a constant.

## 3. Result

The experimental results are obtained after simulation on 70 healthy patients' signals for all 12 leads and using PTB Diagnostic ECG Database (PTBDB). The Physikalisch-Technische Bundesanstalt (PTB) is the National Metrology Institute of Germany. PTB database is provided for PhysioNet and has different morphologies. The ECGs in this database obtain 15 input channels including the conventional 12 leads (i, ii, iii, avr, avl, avf, v1, v2, v3, v4, v5, and v6) together with the 3 Frank lead ECGs (vx, vy, and vz). Input voltage is ±16 mV, input resistance is 100 Ω, ADC resolution is equal to 16 bits with 0.5 *μ*/LSB, and sampling frequency is equal to 1 KHz [20, 21]. The proposed algorithm was performed on a 2.4 GHz Intel core i3 CPU using GNU Octave version 4.0.2 [29]. A selected signal from patient 117 has a variety of physiology and baseline drift. Leads (i, ii, avl, avf, v3, v4, v5, and v6) of record s0291lrem and leads (i, ii, iii, avf, v1, v2, v4, v5, and v6) of record s0292lrem have high amplitude. Leads (i, avl, v2, v3, and v4) of record s0291lrem and leads (avr, avl, and avf) of record s0292lrem have a sharp and tall T-wave.

**Figure 3:**
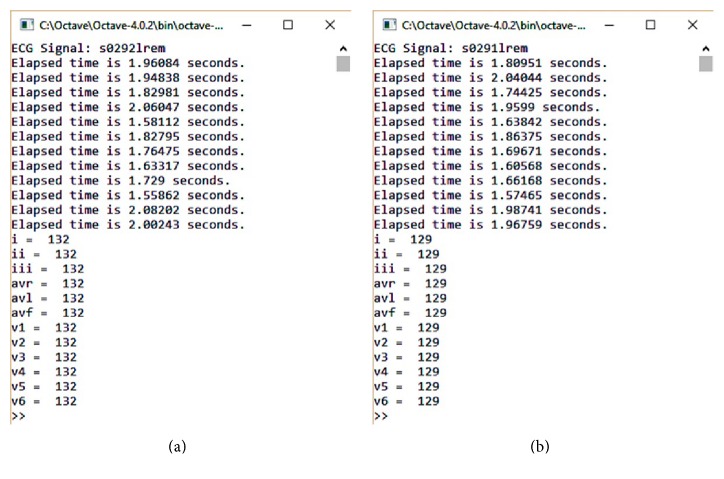
Simulation result. Time and number of peaks detection in each lead are shown. ((a) record s0292lrem; (b) record s0291lrem).

**Figure 4:**
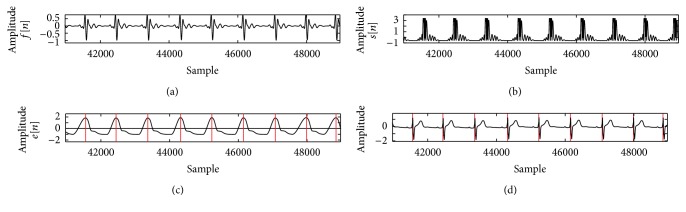
Process of preparations of ECG signal (record s0291lrem, lead v3).

**Figure 5:**
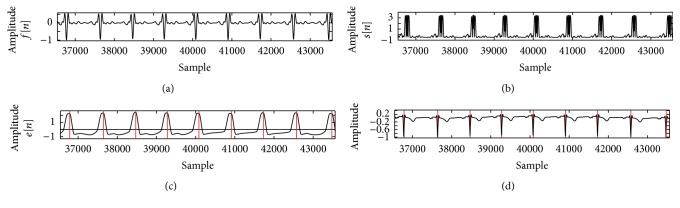
Process of preparation of ECG signal (record s0292lrem, lead avr).

**Figure 6:**
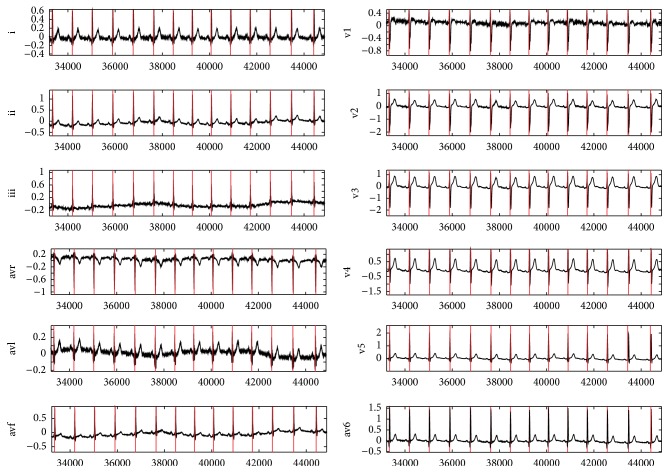
Detected QRS complex of ECG data (record s0292lrem); red line defines QRS complex detection. *y*-axis represents the amplitude, and *x*-axis represents the sample.

**Figure 7:**
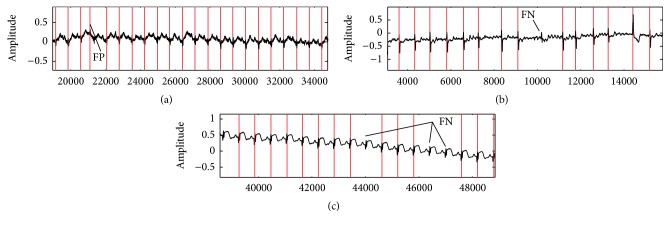
(a) Detected QRS complex of ECG data (record s0020arem, lead avf). Records s0020arem and s0020brem include tall and sharp P-wave and T-wave. In this case, the QRS area has low energy. (b) Detected QRS complex of s0087lrem-lead 3. This case includes Irregular RR interval. (c) Lead v5 of s0089lrem. FN (false negative) is the number of not detected R peaks, and FP (false positive) is the number of noise spikes detected as R peaks. *y*-axis represents the amplitude, and *x*-axis represents the sample.

Figure 3 shows the result of simulation to detect each lead of patient 117 in Octave. Figures 4 and 5 show the process of ECG signal provision and peak detection. The QRS detection of the 12 channels of healthy ECG signal in patient 117 of the PTB database is reported in Table 1 and the Appendix. Detection of the 12 leads is shown in Figure 6.  Figure 7 shows 3 leads of 3 cases.

In order to define performance and efficiency of the algorithm, the Classification Accuracy (Acc), Positive Predictivity (+P), sensitivity (Se), and detection error rate were calculated by using the following equations:

(10) Acc=TPTP+FN+FP×100,+P=TPTP+FP×100,Se=TPTP+FN×100,DER=FP+FNTP×100.

Here, TP defines a true detected peak by the algorithm; FN (false negative) is the number of not detected R peaks, and FP (false positive) is the number of noise spikes detected as R peaks [3, 30].

Figures 4(a) and 5(a) show the output after the band-pass filter *f*[*n*] and normalized amplitude *a*[*n*]. Figures 4(b) and 5(b) show Shannon energy *s*[*n*] and normalized amplitude, and Figures 4(c) and 5(c) show after envelope *e*[*n*] signal. QRS complex of ECG signal is shown in Figures 4(d) and 5(d). Red line defines a detected peak. *y*-axis represents the amplitude, and *x*-axis represents the sample.

In this study, the proposed technique is tested on 840 leads of PTB Diagnostic ECG Database (PTBDB), and values achieved showed that sensitivity (Se) equals 99.924%, detection error rate (DER) equals 0.155%, Positive Predictivity (+P) equals 99.922%, and Classification Accuracy was 99.846%.

## 4. Conclusion

In the present study, the most common methods to remove noise in the ECG signal are evaluated. A Shannon energy-based approach to determine the QRS complex of the 12-lead ECG signal is provided. ECG signal is selected with a variety of physiology from the PTB Database and examined by Octave software. Accuracy and sensitivity achieved from Table 1 showed that the presented algorithm is fast and simple, without complex equations. This algorithm does not need a high memory and high hardware. Diagnosis time for each lead is approximately 2.5 seconds based on Octave. The results showed that algorithm detection has very little lag, less than 0.013 seconds, without error. This lag is generated from stage 3.

## Figures and Tables

**Table 1:** The QRS detection of ECG signal of the PTB database.

Case	TP	FN	FP	DER%	Se%	+P	Acc
s0010_rem	624	0	0	0.000	100.000	100.000	100.000
s0014lrem	1987	0	0	0.000	100.000	100.000	100.000
s0015lrem	1815	0	2	0.110	100.000	99.890	99.890
s0017lrem	1673	0	5	0.299	100.000	99.702	99.702
s0020arem	1906	4	21	1.312	99.791	98.910	98.705
s0020brem	1867	5	20	1.339	99.733	98.940	98.679
s0021arem	2207	1	0	0.045	99.955	100.000	99.955
s0021brem	2196	0	0	0.000	100.000	100.000	100.000
s0025lrem	2382	6	0	0.252	99.749	100.000	99.749
s0029lrem	1638	0	0	0.000	100.000	100.000	100.000
s0031lrem	2111	1	0	0.047	99.953	100.000	99.953
s0035_rem	552	0	0	0.000	100.000	100.000	100.000
s0036lrem	2066	0	2	0.097	100.000	99.903	99.903
s0037lrem	1479	0	3	0.203	100.000	99.798	99.798
s0038lrem	1572	0	0	0.000	100.000	100.000	100.000
s0039lrem	2088	0	0	0.000	100.000	100.000	100.000
s0042lrem	1815	0	0	0.000	100.000	100.000	100.000
s0043lrem	1212	0	0	0.000	100.000	100.000	100.000
s0044lrem	1812	0	0	0.000	100.000	100.000	100.000
s0045lrem	1968	0	0	0.000	100.000	100.000	100.000
s0046lrem	1944	0	0	0.000	100.000	100.000	100.000
s0047lrem	2651	1	0	0.038	99.962	100.000	99.962
s0049lrem	2040	0	0	0.000	100.000	100.000	100.000
s0050lrem	1461	3	0	0.205	99.795	100.000	99.795
s0051lrem	1912	0	2	0.105	100.000	99.896	99.896
s0052lrem	1356	0	0	0.000	100.000	100.000	100.000
s0053lrem	2148	0	0	0.000	100.000	100.000	100.000
s0054lrem	1979	31	2	1.668	98.458	99.899	98.360
s0055lrem	1381	0	1	0.072	100.000	99.928	99.928
s0056lrem	1732	0	0	0.000	100.000	100.000	100.000
s0057lrem	1896	0	0	0.000	100.000	100.000	100.000
s0058lrem	2017	0	1	0.050	100.000	99.950	99.950
s0059lrem	1800	0	0	0.000	100.000	100.000	100.000
s0060lrem	140	0	0	0.000	100.000	100.000	100.000
s0062lrem	1488	0	0	0.000	100.000	100.000	100.000
s0063lrem	1845	3	0	0.163	99.838	100.000	99.838
s0064lrem	1797	3	0	0.167	99.833	100.000	99.833
s0065lrem	1704	0	0	0.000	100.000	100.000	100.000
s0066lrem	1513	0	1	0.066	100.000	99.934	99.934
s0067lrem	424	0	4	0.943	100.000	99.065	99.065
s0068lrem	1377	5	15	1.452	99.638	98.922	98.568
s0069lrem	1188	0	0	0.000	100.000	100.000	100.000
s0070lrem	1983	0	1	0.050	100.000	99.950	99.950
s0071lrem	1848	0	0	0.000	100.000	100.000	100.000
s0072lrem	2040	0	0	0.000	100.000	100.000	100.000
s0073lrem	2125	5	0	0.235	99.765	100.000	99.765
s0074lrem	1140	0	0	0.000	100.000	100.000	100.000
s0075lrem	1453	0	1	0.069	100.000	99.931	99.931
s0076lrem	1308	0	0	0.000	100.000	100.000	100.000
s0077lrem	1692	0	0	0.000	100.000	100.000	100.000
s0078lrem	1225	0	1	0.082	100.000	99.918	99.918
s0079lrem	1620	0	0	0.000	100.000	100.000	100.000
s0080lrem	1556	0	0	0.000	100.000	100.000	100.000
s0082lrem	1602	0	0	0.000	100.000	100.000	100.000
s0083lrem	1465	1	0	0.068	99.932	100.000	99.932
s0084lrem	1464	0	0	0.000	100.000	100.000	100.000
s0085lrem	1276	0	4	0.313	100.000	99.688	99.688
s0097lrem	2133	0	1	0.047	100.000	99.953	99.953
s0101lrem	1500	0	0	0.000	100.000	100.000	100.000
s0103lrem	1273	0	2	0.157	100.000	99.843	99.843
s0149lrem	1572	0	0	0.000	100.000	100.000	100.000
s0152lrem	1532	4	0	0.261	99.740	100.000	99.740
s0087lrem	1654	12	0	0.726	99.280	100.000	99.280
s0088lrem	1728	0	0	0.000	100.000	100.000	100.000
s0091lrem	1380	1	1	0.145	99.928	99.928	99.855
s0095lrem	1797	3	0	0.167	99.833	100.000	99.833
s0096lrem	2603	1	0	0.038	99.962	100.000	99.962
s0150lrem	1583	1	0	0.063	99.937	100.000	99.937
s0090lrem	1358	0	2	0.147	100.000	99.853	99.853
s0093lrem	1249	0	1	0.080	100.000	99.920	99.920
s0291lrem	1548	0	0	0.000	100.000	100.000	100.000
s0292lrem	1584	0	0	0.000	100.000	100.000	100.000
*Total*	*119054*	*91*	*93*	*0.155*	*99.924*	*99.922*	*99.846*

**(a):** 

Leads	s0010_rem			s0014lrem			s0015lrem			s0017lrem		
	TP	FN	FP	TP	FN	FP	TP	FN	FP	TP	FN	FP
i	52	0	0	166	0	0	151	0	0	139	0	0
ii	52	0	0	165	0	0	151	0	0	142	0	3
iii	52	0	0	165	0	0	152	0	1	139	0	0
avr	52	0	0	166	0	0	152	0	0	139	0	0
avl	52	0	0	166	0	0	152	0	1	139	0	0
avf	52	0	0	165	0	0	151	0	0	140	0	1
v1	52	0	0	165	0	0	151	0	0	140	0	1
v2	52	0	0	166	0	0	151	0	0	139	0	0
v3	52	0	0	166	0	0	151	0	0	139	0	0
v4	52	0	0	166	0	0	151	0	0	139	0	0
v5	52	0	0	166	0	0	151	0	0	139	0	0
v6	52	0	0	165	0	0	151	0	0	139	0	0
*Total*	624	0	0	1987	0	0	1815	0	2	1673	0	5

**(b):** 

Leads	s0020arem			s0020brem			s0021arem			s0021brem		
	TP	FN	FP	TP	FN	FP	TP	FN	FP	TP	FN	FP
i	159	0	0	156	0	0	184	0	0	183	0	0
ii	159	0	0	156	0	0	184	0	0	183	0	0
iii	159	0	0	156	0	0	184	0	0	183	0	0
avr	159	0	0	156	0	0	184	0	0	183	0	0
avl	159	0	0	156	0	0	184	0	0	183	0	0
avf	158	3	21	151	5	20	184	0	0	183	0	0
v1	159	0	0	156	0	0	184	0	0	183	0	0
v2	159	0	0	156	0	0	184	0	0	183	0	0
v3	159	0	0	156	0	0	183	1	0	183	0	0
v4	158	1	0	156	0	0	184	0	0	183	0	0
v5	159	0	0	156	0	0	184	0	0	183	0	0
v6	159	0	0	156	0	0	184	0	0	183	0	0
*Total*	1906	4	21	1867	5	20	2207	1	0	2196	0	0

**(c):** 

Leads	s0025lrem			s0029lrem			s0031lrem			s0035_rem		
	TP	FN	FP	TP	FN	FP	TP	FN	FP	TP	FN	FP
i	199	0	0	136	0	0	176	0	0	46	0	0
ii	199	0	0	137	0	0	176	0	0	46	0	0
iii	197	2	0	137	0	0	176	0	0	46	0	0
avr	197	2	0	137	0	0	176	0	0	46	0	0
avl	197	2	0	136	0	0	175	1	0	46	0	0
avf	199	0	0	137	0	0	176	0	0	46	0	0
v1	199	0	0	136	0	0	176	0	0	46	0	0
v2	199	0	0	136	0	0	176	0	0	46	0	0
v3	199	0	0	137	0	0	176	0	0	46	0	0
v4	199	0	0	136	0	0	176	0	0	46	0	0
v5	199	0	0	137	0	0	176	0	0	46	0	0
v6	199	0	0	136	0	0	176	0	0	46	0	0
*Total*	2382	6	0	1638	0	0	2111	1	0	552	0	0

**(d):** 

Leads	s0036lrem			s0037lrem			s0038lrem			s0039lrem		
	TP	FN	FP	TP	FN	FP	TP	FN	FP	TP	FN	FP
i	173	0	1	123	0	0	131	0	0	174	0	0
ii	172	0	0	123	0	0	131	0	0	174	0	0
iii	172	0	0	124	0	1	131	0	0	174	0	0
avr	173	0	1	123	0	0	131	0	0	174	0	0
avl	172	0	0	124	0	1	131	0	0	174	0	0
avf	172	0	0	124	0	1	131	0	0	174	0	0
v1	172	0	0	123	0	0	131	0	0	174	0	0
v2	172	0	0	123	0	0	131	0	0	174	0	0
v3	172	0	0	123	0	0	131	0	0	174	0	0
v4	172	0	0	123	0	0	131	0	0	174	0	0
v5	172	0	0	123	0	0	131	0	0	174	0	0
v6	172	0	0	123	0	0	131	0	0	174	0	0
*Total*	2066	0	2	1479	0	3	1572	0	0	2088	0	0

**(e):** 

Leads	s0042lrem			s0043lrem			s0044lrem			s0045lrem		
	TP	FN	FP	TP	FN	FP	TP	FN	FP	TP	FN	FP
i	152	0	0	101	0	0	151	0	0	164	0	0
ii	151	0	0	101	0	0	151	0	0	164	0	0
iii	151	0	0	101	0	0	151	0	0	164	0	0
avr	152	0	0	101	0	0	151	0	0	164	0	0
avl	152	0	0	101	0	0	151	0	0	164	0	0
avf	151	0	0	101	0	0	151	0	0	164	0	0
v1	151	0	0	101	0	0	151	0	0	164	0	0
v2	151	0	0	101	0	0	151	0	0	164	0	0
v3	151	0	0	101	0	0	151	0	0	164	0	0
v4	151	0	0	101	0	0	151	0	0	164	0	0
v5	151	0	0	101	0	0	151	0	0	164	0	0
v6	151	0	0	101	0	0	151	0	0	164	0	0
*Total*	1815	0	0	1212	0	0	1812	0	0	1968	0	0

**(f):** 

Leads	s0046lrem			s0047lrem			s0049lrem			s0050lrem		
	TP	FN	FP	TP	FN	FP	TP	FN	FP	TP	FN	FP
i	162	0	0	221	0	0	170	0	0	121	1	0
ii	162	0	0	221	0	0	170	0	0	121	1	0
iii	162	0	0	221	0	0	170	0	0	122	0	0
avr	162	0	0	221	0	0	170	0	0	122	0	0
avl	162	0	0	221	0	0	170	0	0	122	0	0
avf	162	0	0	221	0	0	170	0	0	122	0	0
v1	162	0	0	221	0	0	170	0	0	122	0	0
v2	162	0	0	221	0	0	170	0	0	122	0	0
v3	162	0	0	221	0	0	170	0	0	122	0	0
v4	162	0	0	220	1	0	170	0	0	122	0	0
v5	162	0	0	221	0	0	170	0	0	122	0	0
v6	162	0	0	221	0	0	170	0	0	121	1	0
*Total*	1944	0	0	2651	1	0	2040	0	0	1461	3	0

**(g):** 

Leads	s0051lrem			s0052lrem			s0053lrem			s0054lrem		
	TP	FN	FP	TP	FN	FP	TP	FN	FP	TP	FN	FP
i	159	0	0	113	0	0	179	0	0	166	2	0
ii	159	0	0	113	0	0	179	0	0	154	10	2
iii	160	0	0	113	0	0	179	0	0	165	2	0
avr	159	0	0	113	0	0	179	0	0	167	1	0
avl	159	0	0	113	0	0	179	0	0	167	1	0
avf	161	0	2	113	0	0	179	0	0	164	4	0
v1	159	0	0	113	0	0	179	0	0	162	5	0
v2	159	0	0	113	0	0	179	0	0	164	4	0
v3	160	0	0	113	0	0	179	0	0	166	2	0
v4	159	0	0	113	0	0	179	0	0	168	0	0
v5	159	0	0	113	0	0	179	0	0	168	0	0
v6	159	0	0	113	0	0	179	0	0	168	0	0
*Total*	1912	0	2	1356	0	0	2148	0	0	1979	31	2

**(h):** 

Leads	s0055lrem			s0056lrem			s0057lrem			s0058lrem		
	TP	FN	FP	TP	FN	FP	TP	FN	FP	TP	FN	FP
i	115	0	0	144	0	0	158	0	0	168	0	0
ii	116	0	1	144	0	0	158	0	0	168	0	0
iii	115	0	0	145	0	0	158	0	0	168	0	0
avr	115	0	0	145	0	0	158	0	0	168	0	0
avl	115	0	0	144	0	0	158	0	0	168	0	0
avf	115	0	0	145	0	0	158	0	0	168	0	0
v1	115	0	0	144	0	0	158	0	0	168	0	0
v2	115	0	0	144	0	0	158	0	0	168	0	0
v3	115	0	0	145	0	0	158	0	0	169	0	1
v4	115	0	0	144	0	0	158	0	0	168	0	0
v5	115	0	0	144	0	0	158	0	0	168	0	0
v6	115	0	0	144	0	0	158	0	0	168	0	0
*Total*	1381	0	1	1732	0	0	1896	0	0	2017	0	1

**(i):** 

Leads	s0059lrem			s0060lrem			s0090lrem			s0062lrem		
	TP	FN	FP	TP	FN	FP	TP	FN	FP	TP	FN	FP
i	150	0	0	140	0	0	113	0	0	124	0	0
ii	150	0	0	140	0	0	113	0	0	124	0	0
iii	150	0	0	140	0	0	113	0	0	124	0	0
avr	150	0	0	140	0	0	113	0	0	124	0	0
avl	150	0	0	140	0	0	113	0	0	124	0	0
avf	150	0	0	140	0	0	113	0	0	124	0	0
v1	150	0	0	140	0	0	113	0	0	124	0	0
v2	150	0	0	140	0	0	113	0	0	124	0	0
v3	150	0	0	140	0	0	114	0	1	124	0	0
v4	150	0	0	140	0	0	114	0	1	124	0	0
v5	150	0	0	140	0	0	113	0	0	124	0	0
v6	150	0	0	140	0	0	113	0	0	124	0	0
*Total*	1800	0	0	1680	0	0	1358	0	2	1488	0	0

**(j):** 

Leads	s0063lrem			s0064lrem			s0065lrem			s0066lrem		
	TP	FN	FP	TP	FN	FP	TP	FN	FP	TP	FN	FP
i	154	0	0	149	1	0	142	0	0	126	0	0
ii	151	3	0	150	0	0	142	0	0	127	0	1
iii	154	0	0	150	0	0	142	0	0	126	0	0
avr	154	0	0	150	0	0	142	0	0	126	0	0
avl	154	0	0	149	1	0	142	0	0	126	0	0
avf	154	0	0	150	0	0	142	0	0	126	0	0
v1	154	0	0	150	0	0	142	0	0	126	0	0
v2	154	0	0	150	0	0	142	0	0	126	0	0
v3	154	0	0	150	0	0	142	0	0	126	0	0
v4	154	0	0	150	0	0	142	0	0	126	0	0
v5	154	0	0	150	0	0	142	0	0	126	0	0
v6	154	0	0	149	1	0	142	0	0	126	0	0
*Total*	1845	3	0	1797	3	0	1704	0	0	1513	0	1

**(k):** 

Leads	s0067lrem			s0068lrem			s0069lrem			s0070lrem		
	TP	FN	FP	TP	FN	FP	TP	FN	FP	TP	FN	FP
i	35	0	0	115	0	0	99	0	0	165	0	0
ii	35	0	0	115	0	0	99	0	0	165	0	0
iii	36	0	1	113	2	11	99	0	0	165	0	0
avr	35	0	0	115	0	0	99	0	0	165	0	0
avl	36	0	1	116	0	2	99	0	0	165	0	0
avf	36	0	1	115	0	0	99	0	0	165	0	0
v1	35	0	0	116	0	2	99	0	0	165	0	0
v2	35	0	0	114	1	0	99	0	0	167	0	1
v3	36	0	1	115	0	0	99	0	0	166	0	0
v4	35	0	0	114	1	0	99	0	0	165	0	0
v5	35	0	0	114	1	0	99	0	0	165	0	0
v6	35	0	0	115	0	0	99	0	0	165	0	0
*Total*	424	0	4	1377	5	15	1188	0	0	1983	0	1

**(l):** 

Leads	s0071lrem			s0072lrem			s0073lrem			s0074lrem		
	TP	FN	FP	TP	FN	FP	TP	FN	FP	TP	FN	FP
i	154	0	0	170	0	0	177	0	0	95	0	0
ii	154	0	0	170	0	0	177	0	0	95	0	0
iii	154	0	0	170	0	0	173	5	0	95	0	0
avr	154	0	0	170	0	0	178	0	0	95	0	0
avl	154	0	0	170	0	0	177	0	0	95	0	0
avf	154	0	0	170	0	0	177	0	0	95	0	0
v1	154	0	0	170	0	0	178	0	0	95	0	0
v2	154	0	0	170	0	0	178	0	0	95	0	0
v3	154	0	0	170	0	0	178	0	0	95	0	0
v4	154	0	0	170	0	0	177	0	0	95	0	0
v5	154	0	0	170	0	0	177	0	0	95	0	0
v6	154	0	0	170	0	0	178	0	0	95	0	0
*Total*	1848	0	0	2040	0	0	2125	5	0	1140	0	0

**(m):** 

Leads	s0075lrem			s0076lrem			s0077lrem			s0078lrem		
	TP	FN	FP	TP	FN	FP	TP	FN	FP	TP	FN	FP
i	121	0	0	109	0	0	141	0	0	102	0	0
ii	121	0	0	109	0	0	141	0	0	103	0	1
iii	121	0	0	109	0	0	141	0	0	102	0	0
avr	121	0	0	109	0	0	141	0	0	102	0	0
avl	121	0	0	109	0	0	141	0	0	102	0	0
avf	121	0	0	109	0	0	141	0	0	102	0	0
v1	122	0	1	109	0	0	141	0	0	102	0	0
v2	121	0	0	109	0	0	141	0	0	102	0	0
v3	121	0	0	109	0	0	141	0	0	102	0	0
v4	121	0	0	109	0	0	141	0	0	102	0	0
v5	121	0	0	109	0	0	141	0	0	102	0	0
v6	121	0	0	109	0	0	141	0	0	102	0	0
*Total*	1453	0	1	1308	0	0	1692	0	0	1225	0	1

**(n):** 

Leads	s0079lrem			s0080lrem			s0093lrem			s0082lrem		
	TP	FN	FP	TP	FN	FP	TP	FN	FP	TP	FN	FP
i	135	0	0	130	0	0	104	0	0	133	0	0
ii	135	0	0	129	0	0	104	0	0	133	0	0
iii	135	0	0	130	0	0	104	0	0	134	0	0
avr	135	0	0	130	0	0	104	0	0	134	0	0
avl	135	0	0	130	0	0	104	0	0	133	0	0
avf	135	0	0	129	0	0	105	0	1	133	0	0
v1	135	0	0	129	0	0	104	0	0	134	0	0
v2	135	0	0	129	0	0	104	0	0	133	0	0
v3	135	0	0	130	0	0	104	0	0	134	0	0
v4	135	0	0	130	0	0	104	0	0	134	0	0
v5	135	0	0	130	0	0	104	0	0	134	0	0
v6	135	0	0	130	0	0	104	0	0	133	0	0
*Total*	1620	0	0	1556	0	0	1249	0	1	1602	0	0

**(o):** 

Leads	s0083lrem			s0084lrem			s0085lrem			s0097lrem		
	TP	FN	FP	TP	FN	FP	TP	FN	FP	TP	FN	FP
i	122	0	0	122	0	0	106	0	0	178	0	1
ii	123	1	0	122	0	0	106	0	0	177	0	0
iii	122	0	0	122	0	0	108	0	2	178	0	0
avr	122	0	0	122	0	0	106	0	0	178	0	0
avl	122	0	0	122	0	0	106	0	0	178	0	0
avf	122	0	0	122	0	0	106	0	0	178	0	0
v1	122	0	0	122	0	0	106	0	0	177	0	0
v2	122	0	0	122	0	0	108	0	2	178	0	0
v3	122	0	0	122	0	0	106	0	0	178	0	0
v4	122	0	0	122	0	0	106	0	0	178	0	0
v5	122	0	0	122	0	0	106	0	0	178	0	0
v6	122	0	0	122	0	0	106	0	0	177	0	0
*Total*	1465	1	0	1464	0	0	1276	0	4	2133	0	1

**(p):** 

Leads	s0101lrem			s0103lrem			s0149lrem			s0152lrem		
	TP	FN	FP	TP	FN	FP	TP	FN	FP	TP	FN	FP
i	125	0	0	106	0	1	131	0	0	128	0	0
ii	125	0	0	106	0	0	131	0	0	128	0	0
iii	125	0	0	107	0	1	131	0	0	128	0	0
avr	125	0	0	106	0	0	131	0	0	128	0	0
avl	125	0	0	106	0	0	131	0	0	128	0	0
avf	125	0	0	106	0	0	131	0	0	124	4	0
v1	125	0	0	106	0	0	131	0	0	128	0	0
v2	125	0	0	106	0	0	131	0	0	128	0	0
v3	125	0	0	106	0	0	131	0	0	128	0	0
v4	125	0	0	106	0	0	131	0	0	128	0	0
v5	125	0	0	106	0	0	131	0	0	128	0	0
v6	125	0	0	106	0	0	131	0	0	128	0	0
*Total*	1500	0	0	1273	0	2	1572	0	0	1532	4	0

**(q):** 

Leads	s0087lrem			s0088lrem			s0089lrem			s0091lrem		
	TP	FN	FP	TP	FN	FP	TP	FN	FP	TP	FN	FP
i	142	0	0	144	0	0	196	0	0	114	1	0
ii	138	4	0	144	0	0	194	2	0	115	0	0
iii	136	6	0	144	0	0	196	0	0	115	0	0
avr	138	0	0	144	0	0	196	0	0	115	0	0
avl	138	0	0	144	0	0	196	0	0	115	0	0
avf	137	1	0	144	0	0	196	0	0	116	0	1
v1	136	0	0	144	0	0	196	0	0	115	0	0
v2	138	0	0	144	0	0	196	0	0	115	0	0
v3	137	1	0	144	0	0	196	0	0	115	0	0
v4	138	0	0	144	0	0	196	0	0	115	0	0
v5	138	0	0	144	0	0	156	40	0	115	0	0
v6	138	0	0	144	0	0	196	0	0	115	0	0
*Total*	1654	12	0	1728	0	0	2310	42	0	1380	1	1

**(r):** 

Leads	s0095lrem			s0096lrem			s0150lrem			s0150lrem		
	TP	FN	FP	TP	FN	FP	TP	FN	FP	TP	FN	FP
i	149	1	0	217	0	0	0	0	0	132	0	0
ii	150	0	0	217	0	0	0	0	0	132	0	0
iii	150	0	0	217	0	0	0	0	0	132	0	0
avr	150	0	0	217	0	0	0	0	0	132	0	0
avl	150	0	0	217	0	0	0	0	0	132	0	0
avf	150	0	0	217	0	0	0	0	0	132	0	0
v1	149	1	0	217	0	0	0	0	0	132	0	0
v2	149	1	0	217	0	0	0	0	0	131	1	0
v3	150	0	0	217	0	0	0	0	0	132	0	0
v4	150	0	0	217	0	0	0	0	0	132	0	0
v5	150	0	0	216	1	0	0	0	0	132	0	0
v6	150	0	0	217	0	0	0	0	0	132	0	0
*Total*	1797	3	0	2603	1	0	0	0	0	1583	1	0

**(s):** 

Leads	s0291lrem			s0292lrem		
	TP	FN	FP	TP	FN	FP
i	129	0	0	132	0	0
ii	129	0	0	132	0	0
iii	129	0	0	132	0	0
avr	129	0	0	132	0	0
avl	129	0	0	132	0	0
avf	129	0	0	132	0	0
v1	129	0	0	132	0	0
v2	129	0	0	132	0	0
v3	129	0	0	132	0	0
v4	129	0	0	132	0	0
v5	129	0	0	132	0	0
v6	129	0	0	132	0	0
*Total*	1548	0	0	1584	0	0

## Appendix

See Table 2.
